# Complete Genome Sequence of a Chemolithoautotrophic Iron-Oxidizing Bacterium, Acidithiobacillus ferrooxidans Strain NFP31, Isolated from Volcanic Ash Deposits on Miyake-Jima, Japan

**DOI:** 10.1128/mra.01006-21

**Published:** 2022-01-13

**Authors:** Tatsuhiro Kato, Yong Guo, Reiko Fujimura, Takamichi Nakamura, Tomoyasu Nishizawa, Yasuro Kurusu, Hiroyuki Ohta

**Affiliations:** a Graduate School of Agriculture, Ibaraki University, Ami, Ibaraki, Japan; b Ibaraki University College of Agriculture, Ami, Ibaraki, Japan; c Kumagai Gumi, Tsukuba, Ibaraki, Japan; Universidad Nacional Autónoma de México

## Abstract

The genome sequence of Acidithiobacillus ferrooxidans strain NFP31, which is a chemolithoautotrophic iron-oxidizing bacterium that inhabits acidified volcanic deposits on Mount Oyama, Miyake Island (Miyake-jima), Japan, was determined to identify the genetic characteristics associated with pioneer microbes in newly placed pyroclastic deposits.

## ANNOUNCEMENT

Acidithiobacillus ferrooxidans, mainly derived from acid mine drainage, can oxidize inorganic compounds such as ferrous iron, elemental sulfur, reduced sulfur compounds, and hydrogen under acidic conditions (pH 2.0 to 3.5) (1–3); however, only a few completed genomes are available for this species to date. Here, we sequenced the whole genome of strain NFP31, which was predicted to be *A. ferrooxidans* based on the 16S rRNA sequence, obtained from recent volcanic ash deposits (pH 3.4 to 3.9) on Miyake-jima (34°05′N, 139°31′E), profiting from a predominance of chemolithoautotrophic bacteria in volcanic deposits from the eruption that occurred in 2000 (4–6).

Strain NFP31 genomic DNA was extracted from cells grown in modified 9K medium (pH 2.0) (7), which contained KH_2_PO_4_ instead of K_2_HPO_4_, at 30°C for 10 days, using a DNeasy PowerSoil kit (Qiagen). A nanopore sequencing library was prepared using a ligation sequencing kit (SQK-LSK109; Oxford Nanopore Technologies) and sequenced on a PromethION platform to generate 1.69 Gb of long reads. Base calling and adapter-sequence removal were performed using Guppy (8) and Porechop, respectively. High-quality reads were selected using NanoFilt (9). Additionally, a paired-end library was prepared using an MGIEasy PCR-free DNA library prep set (MGI) and sequenced on an MGI DNBSEQ platform. Adapter-sequence removal and read filtering were performed using Cutadapt (10). Finally, a total of 33,125 long reads (average length, 19,405 bp) and 3,439,964 paired-end short reads (2 × 150 bp) were obtained from the Nanopore and MGISEQ libraries, respectively. The Nanopore reads were assembled using Trycycler (11) to produce two circular contigs, which were subsequently polished using medaka, and the Burrows-Wheeler Aligner (BWA) (12) and Pilon (13) were used with the short-read data set. The completed genome was identified phylogenomically using JSpeciesWS (14), and functional annotation was carried out using DFAST (15) and KofamKOALA (16), with the basic local alignment search tool (BLAST) (17) used for manual annotation. The software version and option settings used are shown in Table S1 posted at https://doi.org/10.6084/m9.figshare.16684999.

The complete genome sequence of strain NFP31 consists of a 3,205,477-bp circular chromosome with a GC content of 58.5% and a 44,377-bp circular plasmid with 56.5% GC content. The NFP31 chromosome comprised 3,266 predicted protein-coding sequences (CDSs), 6 rRNAs, 47 tRNAs, and 1 transfer-messenger RNA (tmRNA), and the plasmid contained 52 predicted CDSs. As shown in Table 1, biogeochemical cycle genes related to nitrogen fixation, Calvin-Benson cycling carbon fixation, iron-sulfur oxidation, and hydrogen oxidation were found in the NFP31 chromosome. To assess the species definition, whole-genome average nucleotide identity analysis was examined using complete-genome sequences of *A. ferrooxidans* strains ATCC 23270^T^ (18), ATCC 53993 (GenBank accession number CP001132), and YNTRS-40, which possessed a circular plasmid isolated from a hot spring (pH 1.0 to 4.5) (19), and showed 98.91%, 99.09%, and 99.99% sequence similarity, respectively, with strain NFP31. The gene features of the acidophilic bacterium *A. ferrooxidans* strain NFP31 represent an important factor to initiate ecosystem development in Miyake-jima early volcanic deposits.

**TABLE 1:** Genes involved in biogeochemical cycling of *A. ferrooxidans* strain NFP31

Locus tag	Biogeochemical cycling/product (gene name)
Carbon fixation	
ANFP_19520	Ribulose bisphosphate carboxylase (*cbbM*)
ANFP_22090, ANFP_30260	Ribulose bisphosphate carboxylase large subunit (*cbbL*)
ANFP_22100, ANFP_30270	Ribulose bisphosphate carboxylase small subunit (*rbcS*)
Nitrogen fixation	
ANFP_15240	Nitrogenase-stabilizing protein (*nifW*)
ANFP_15270, ANFP_15550	Cysteine desulfurase (*nifS*)
ANFP_15280	Nitrogen fixation protein (*nifU*)
ANFP_15310	Nitrogen fixation protein (*nifQ*)
ANFP_15350	Nitrogen fixation protein (*nifX*)
ANFP_15360	Nitrogenase iron-molybdenum cofactor biosynthesis protein (*nifN*)
ANFP_15370	Nitrogenase iron-molybdenum cofactor biosynthesis protein (*nifE*)
ANFP_15400	Nitrogenase molybdenum-iron protein beta chain (*nifK*)
ANFP_15410	Nitrogenase molybdenum-iron protein alpha chain (*nifD*)
ANFP_15420	Nitrogenase iron protein (*nifH*)
ANFP_15460	Nif-specific transcriptional activator (*nifA*)
ANFP_15470	FeMo cofactor biosynthesis (*nifB*)
ANFP_15530	NifZ protein (*nifZ*)
ANFP_15560	Putative nitrogen fixation protein (*nifT*)
Iron-sulfur oxidation	
ANFP_08020	Ubiquinol oxidase subunit 2 (*cyoA*)
ANFP_08030	Ubiquinol oxidase subunit 1 (*cyoB*)
ANFP_08040	Ubiquinol oxidase subunit 3 (*cyoC*)
ANFP_12530	Cytochrome *d* ubiquinol oxidase subunit II (*cydB*)
ANFP_12540	Cytochrome *d* ubiquinol oxidase subunit I
ANFP_16060, ANFP_30810	Pyridine nucleotide-disulfide oxidoreductase (*sqr*)
ANFP_27230, ANFP_30730	YciK family oxidoreductase (*yciK*)
ANFP_27240	Ubiquinol-cytochrome *c* reductase iron-sulfur subunit (*petA*)
ANFP_27250	Cytochrome *b* (*petB*)
ANFP_27260	Cytochrome *c* (*petC*)
ANFP_30720	C-type cytochrome (*cyc*)
ANFP_30750	Cytochrome *c* biogenesis protein
ANFP_30760	Cytochrome *c* biogenesis protein
ANFP_31070	Rusticyanin
ANFP_31110	Cytochrome *c* oxidase subunit I (*ctaD*)
ANFP_31120	Cytochrome *c* oxidase (*coxM*)
Hydrogen oxidation	
ANFP_08710	Hydrogenase transcriptional regulatory protein (*hoxA*)
ANFP_08720	Hydrogenase
ANFP_08730	Cytochrome *c*_3_ hydrogenase
ANFP_08760	Hydrogenase maturation protease
ANFP_08840	Hydrogenase assembly protein (*hypC*)
ANFP_08860, ANFP_32500	Hydrogenase maturation factor (*hypD*)
ANFP_08870	Hydrogenase expression/formation protein (*hypE*)
ANFP_08880, ANFP_32470	Hydrogenase nickel incorporation protein (*hypA*)
ANFP_08890	Hydrogenase accessory protein (*hypB*)
ANFP_12370	Hydrogenase (*vhtD*)
ANFP_12380	Ni/Fe hydrogenase subunit alpha (*hydA*)
ANFP_12390	Sulfhydrogenase subunit delta (*hydD*)
ANFP_12400	Ni/Fe hydrogenase subunit gamma (*hydG*)
ANFP_19460	Hydrogenase 4 subunit B (*hyfB*)
ANFP_19470	Formate hydrogenlyase subunit 4 (*hyfC*)
ANFP_19490	Hydrogenase 4 subunit F (*hyfF*)
ANFP_19500	Hydrogenase expression protein (*hypE*)
ANFP_19510	Hydrogenase
ANFP_32410	Membrane protein (*hyaP*)
ANFP_32430	Hydrogenase small subunit
ANFP_32460	Hydrogenase 2 large subunit
ANFP_32490	Hydrogenase assembly protein (*hypC*)

### Data availability.

The genome sequences of *A. ferrooxidans* strain NFP31 were deposited in DDBJ/ENA/GenBank under the accession numbers AP025160 and AP025161 for the circular chromosome and plasmid, respectively. The raw read data set is available under SRA number DRA012631.
